# Five‐Year (2017–2022) Evolutionary Dynamics of Human Coronavirus HKU1 in Southern France With Emergence of Viruses Harboring Spike H512R Substitution

**DOI:** 10.1002/jmv.70217

**Published:** 2025-02-14

**Authors:** Houmadi Hikmat, Lorlane Le Targa, Céline Boschi, Justine Py, Aurélie Morand, Jean‐Christophe Lagier, Sarah Aherfi, Jacques Fantini, Bernard La Scola, Philippe Colson

**Affiliations:** ^1^ Microbes Evolution Phylogeny and Infections (MEPHI), Aix‐Marseille Université (AMU) Marseille France; ^2^ IHU Méditerranée Infection Marseille France; ^3^ Biosellal Lyon France; ^4^ Assistance Publique‐Hôpitaux de Marseille (AP‐HM) Marseille France; ^5^ Service d'accueil des Urgences Pédiatriques, Hôpital Nord Assistance Publique‐Hôpitaux de Marseille (AP‐HM) Marseille France; ^6^ Service de Pédiatrie Générale, Hôpital Timone Assistance Publique‐Hôpitaux de Marseille (AP‐HM) Marseille France; ^7^ INSERM UMR UA 16, Aix‐Marseille Université Marseille France

**Keywords:** coronavirus, evolution, genome, HKU1, molecular epidemiology, respiratory virus, TMPRSS2

## Abstract

HCoV‐HKU1 diversity and evolution were scarcely studied. We performed next‐generation sequencing (NGS) and analysis of HCoV‐HKU1 genomes over 5 years. NGS used Illumina technology on NovaSeq 6000 following whole genome PCR amplification by an in‐house set of primers designed using Gemi and PrimalScheme. Genome assembly and analyses used CLC Genomics, Mafft, BioEdit, Nextstrain, Nextclade, MEGA, and iTol bioinformatic tools. Spike molecular modeling and dynamics simulations used Molegro Molecular Viewer and Hyperchem programs. Twenty‐eight PCR systems allowed obtaining 158 HCoV‐HKU1 genomes including 69 and 89 of genotypes A and B, respectively. Both genotypes co‐circulated during the study period but one predominated each year. A total of 1683 amino acid substitutions including 80 in ≥ 10 genomes were detected in genotype A relatively to a 2004 reference. H512R in spike, first detected in 2009 and reported as involved in antibody neutralization, was found in all genotype A, almost always with V387I and K478N, and was predicted here to significantly improve cellular TMPRSS2 protein binding. Also, 1802 amino acid substitutions including 64 in ≥ 10 genomes were detected in genotype B relatively to a 2005 reference. This study substantially expands the global set of HCoV‐HKU1 genomes. Genomics with protein structural analyses contributed to our understanding of HCoV‐HKU1 evolution.

## 1 Introduction

Seven coronaviruses are currently known to cause respiratory infections in humans, of which four (human coronaviruses [HCoV]‐OC43, 229E, NL63, and HKU1) are endemic [1, 2]. Coronaviruses have a wide host spectrum including humans and nonhuman animals, and are capable of crossing the species barriers, such as for the case of HCoV‐HKU1 that originated from a murine host [3]. HCoV‐HKU1 was discovered in China in 2004 in a 71‐year‐old patient with comorbidities who exhibited cough and fever [4]. This virus belongs to the *Betacoronavirus* genus. The viral genome is approximately 30 kilobase–long, and its two‐thirds consist of ORF1a and 1b, which encode the set of nonstructural proteins (NSP) 1–16. The remaining third of the genome encodes the structural proteins, namely the spike, the membrane glycoprotein, the viral envelope, and the nucleocapsid. One of the characteristics of HCoV‐HKU1 is the presence of a gene encoding a hemagglutinin esterase (HE), as for the case of some betacoronaviruses among which HCoV‐OC43, but which is absent in alphacoronavirus genomes. Three genotypes of HCoV‐HKU1, named A, B, and C, have been described [4, 5]. The protein receptor for HCoV‐HKU1 is still unknown, but the viral spike binds to 9‐O‐acetylated sialic acid of the host cell, and HKU1 is thought to have TMPRSS2 as a receptor involved in fusion [6, 7].

HCoV‐HKU1 is responsible for infections of the upper respiratory tract in most cases, but can also affect the lower respiratory tract [8, 9]. Symptomatology is variable and comparable to those of other respiratory viruses, with such symptoms as fever, cough, rhinitis, and dyspnea [4, 10]. The seasonality of this virus varies depending on the geographical area. In temperate countries such as France, the virus is mainly detected during winter, and spring [11, 12, 13].

Unlike other viruses such as SARS‐CoV‐2 and influenza viruses, there are few evolutionary studies reported for HCoV‐HKU1. We believe that understanding the dynamics of HCoV‐HKU1 infections requires an understanding of the genetic evolution of these viruses, but as of December 4, 2023, there were only 106 complete genomes (between 29 367 and 30 144 nucleotides in size) of HCoV‐HKU1 in the NCBI GenBank nucleotide sequence database [14] (https://www.ncbi.nlm.nih.gov/genbank/), which is very few with regard to genomic and genotypic surveillance, and dramatically low compared to the numbers of SARS‐CoV‐2 complete genomes in GenBank and in GISAID which are more than 8 and 15 millions, respectively [15, 16]. Therefore, here we aimed to carry out retrospectively the sequencing and analysis of HCoV‐HKU1 genomes from samples of patients diagnosed as HCoV‐HKU1 RNA‐positive by real‐time PCR (qPCR) during the period 2017–2022 at the university and public hospitals of Marseille, southern France.

## 2 Materials and Methods

### 2.1 Respiratory Samples

Nasopharyngeal samples analyzed retrospectively were sent to the clinical microbiology‐virology laboratory of university and public hospitals of Marseille, southeastern France, for routine clinical diagnosis of respiratory virus infections. They were collected over a 67 month‐period that extends from February 2017 to August 2022. RNA was extracted from nasopharyngeal samples using the KingFisher Flex system (Thermo Fisher Scientific, Waltham, MA, USA), following the manufacturer's instructions. HCoV‐HKU1 was diagnosed by real‐time reverse transcription‐PCR (qPCR) using either the FTD Respiratory pathogens 21 assay (Fast Track Diagnosis, Luxembourg) or the BioFire FilmArray Respiratory panel 2 plus assay (Biomérieux, Marcy‐l'Etoile, France). The present study has been registered on the Health Data Access Portal of Marseille public and university hospitals (Assistance Publique‐Hôpitaux de Marseille [AP‐HM]) with No. PADS24‐190 and was approved by the Ethics and Scientific Committee of AP‐HM.

### 2.2 Next‐Generation Sequencing (NGS)

#### 2.2.1 Primer Design for PCR Amplification of Overlaping Regions Covering the Whole Viral Genome

All HCoV‐HKU1 complete genomes available in Genbank (https://www.ncbi.nlm.nih.gov/genbank/) were collected in April 2022. They were aligned using the Mafft software [17]. Primers targeting the regions the most conserved within the obtained alignment were designed using the GEMI software, which allowed us to choose amplicon size (447–1587 nucleotides), primer size (19–23 nucleotides), temperature of the PCR hybridization step (58°C–60°C), and maximum number of degenerated nucleotide positions (1–4) to handle genetic diversity in the set of genomes [18]. The PrimalScheme software (https://primalscheme.com/), which previously allowed designing the mutiplex PCR systems for SARS‐CoV‐2 (‘ARTIC’,https://artic.network/ncov-2019) and Zika virus [19], was also used here. Unlike GEMI, PrimalScheme only enables the choice of amplicon size.

#### 2.2.2 PCR Amplification of Overlaping Regions Covering the Whole Genomes and NGS

The KingFisher Flex system (Thermo Fisher Scientific, Waltham, MA, USA) was used to extract viral RNA from the residual nasopharyngeal samples, following the manufacturer's instructions. Two pools were produced by mixing the previously designed PCR primers following an “Artic‐like” methodology priorly implemented for Zika virus [20] and SARS‐CoV‐2 (https://artic.network/). Reverse transcription‐PCR amplification was performed using the SuperScript III One‐Step with High‐Fidelity Platinum Taq DNA Polymerase kit in a reaction containing 12.5 µL of 2× mix, 0.7 µL of enzyme, 0.75 µL of each PCR pool, 3 µL of nucleic acid extract, and 8.05 µL of water. The PCR program used included a reverse transcription step at 50°C for 25 min, then an initial denaturation step at 95°C for 2 min, followed by 39 PCR cycles comprising a denaturation step at 95°C for 15 s, a primer hybridization step at 58°C for 45 s, and an elongation step at 70°C for 2 min, before a final elongation step at 70°C for 5 min. Purification of the amplicons was performed on a NucleoFast 96‐well plate (Macherey Nagel ref 743100.50, Hoerdt, France); elution was carried out with 40 µL of pure water. Once purified, the amplicons generated from the two pools of PCR systems were mixed together.

For the setting up of our system, NGS was performed with the Oxford Nanopore Technology using the Ligation Sequencing Kit (SQK‐LSK109) library on a SpotON flow cell Mk I, R9.4.1 and a GridION instrument, following the manufacturer's instructions (Oxford Nanopore Technologies, Oxford, UK). Thereafter, at the step of using our ready to use “Artic‐like” system on the studied nasopharyngeal samples, NGS was performed with the Illumina technology on a NovaSeq 6000 instrument (Illumina Inc., San Diego, CA, USA) using the COVIDSeq protocol but replacing the COVID‐19 ARTIC PCR primers by the PCR primers designed here. Loading of the NovaSeq 6000 instrument was performed on an SP flow cell according to the NovaSeq‐XP workflow with a reading of 2 × 50, following a previously described procedure [21]. The HCoV‐HKU1 genomes obtained were submitted to the GenBank database (https://www.ncbi.nlm.nih.gov/genbank/; GenBank Accession no: PP356751 ‐ PP356908).

### 2.3 Bioinformatic Analyses of Viral Genomes

The CLC Genomics Workbench v.7 software was used for the bioinformatics analysis of our sequences. A trimming step was first performed followed by the mapping of NGS reads against the HCoV‐HKU1 reference genomes GenBank Accession no. NC_006577.2 and LC315651.2, that was performed with as minimum thresholds a coverage of 80% of reads on the reference genome and a nucleotide identity of 90%. All obtained consensus genomes with at least 80% coverage of the reference genome and a sequencing depth of at least three were recovered. The Mafft software [17] was used for sequence alignments and the MEGA v.11 software [22] was used to perform phylogeny reconstruction using the Neighbor‐Joining and Maximum composite likelihood parameter methods with 1000 replicates. Phylogenetic analysis was performed by including, in our data set of sequences, genomes available in GenBank that belong to the three HCoV‐HKU1 genotypes A, B, and C. iTOL [23] was used to visualize phylogenetic trees. The Nextclade tool (https://clades.nextstrain.org/) [24] was adapted to be able identifying the mutations and the different lineages to which the genomes we obtained belong. Nucleotide and amino acid diversities as well as mutational patterns were determined in obtained genomes according to the viral genotype, relative to two reference genomes of genotypes A and B. For each genotype, an historical reference HCoV‐HKU1 genome, NC_006577.2 for genotype A and DQ415911.1 for genotype B, dating back to 2004 and 2005, respectively, was used. In addition, more recent reference genomes, LC315650.2 for genotype A and LC315651.2 for genotype B, dating back to 2014 and 2016, respectively, were used. We used the same methodology than in a previous study [25] to classify genes into “structural,” “informational,” “other nonstructural” and “accessory” genes (Supporting Information S1: Methods).

### 2.4 Molecular Modeling of the HCoV‐HKU1 Spike

The coordinates of HCoV‐HKU1 spike proteins (both monomers and trimers) were retrieved from pdb 8ohn (open conformation) and 8opn (closed conformation) [26]. The structure of human TMPRSS2 was retrieved from pdb 7 meq. The structure of the TMPRSS2‐HKU1 complex was retrieved from pdb 8vgt. The models were visualized with the Molegro Molecular Viewer (http://molexus.io/molegro-molecular-viewer), as previously described [27]. Energy‐minimized models were generated with the Polak‐Ribiere algorithm and with the Bio‐CHARMM force field in Hyperchem using a maximum of 3 × 10^5^ steps and a root mean square gradient of 0.01 kcal/mol・Å as the convergence condition [28]. Mutations were introduced with Deep View/Swiss‐Pdb viewer [29], followed by several rounds of energy minimization as described previously [30, 31]. Molecular dynamics simulations (MDS) were performed on a Dell workstation with the HyperChem program (http://www.hypercubeusa.com), as previously described; simulation time was 100 ns [32]. The systems were equilibrated at constant temperature (310 K) and constant pressure (1 atm) [28]. The simulation time was sufficient to reach a root mean square deviation < 2 Å due to the reliability of starting conditions generated by energy minimization of the Protein DataBank files obtained from experimental studies. Protein visualization and energy of interaction calculations were obtained from the Molegro software [27].

## 3 Results

### 3.1 Primer Design for PCR Amplification of Overlaping Regions Covering the Whole HCoV‐HKU1 Genomes

For primer design, the Gemi software generated 156 PCR primer pairs. Of these 156, 36 were tested, and 25 were selected as they provided PCR amplicons in sufficient amounts and of appropriate size as assessed by gel agarose electrophoresis. Three primer pairs designed using the PrimalScheme software were added. Finally, 28 PCR systems covering the genome were selected and used (Table S1). Those showing weak bands on a 1.5% agarose gel were optimized by progressively increasing the primer concentrations. These latter ranged between 10 and 15 pmol/µL. Two pools were then made with these 28 primer systems not to have unplanned hybridization during PCR. The Oxford Nanopore technology and a Gridion instrument were used for testing and optimizing the PCR amplifications, then all processed samples were sequenced using the Illumina technology on a NovaSeq 6000 instrument.

### 3.2 NGS

**Figure 1:**
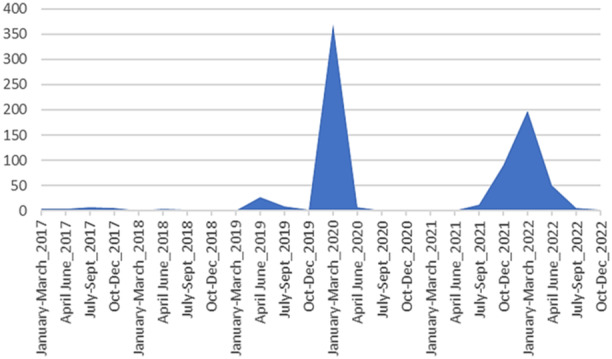
Temporal distribution of HCoV‐HKU1 samples used for next‐generation sequencing between 2017 and 2022.

The whole HCoV‐HKU1 nucleic acid enrichment and NGS strategy were applied to RNA extracts recovered from HCoV‐HKU1 RNA‐positive nasopharyngeal samples. A total of 803 nasopharyngeal samples, which were tested in the diagnosis setting in our institute between January 2017 and December 2022 by PCR assays targeting respiratory viruses other than SARS‐CoV‐2, were positive for HCoV‐HKU1. In 2020 and 2022, the HCoV‐HKU1 epidemic peaks occurred between January and March, while no HCoV‐KHU1 infection was diagnosed between July 2020 and June 2021 (Figure 1). Among the 803 HCoV‐HKU1‐positive samples, we could retrieve 411 from our freezers (−80°C storage) and all their RNA extracts were submited to the ARTIC‐like amplification with our PCR primers’ pools. The cycle threshold (Ct) of the qPCR used to diagnose HCoV‐HKU1 infection was known for 293 of these 411 samples and ranged between 13 and 36 with a mean (± standard deviation) value of 24.8 ± 5.2. For these 411 samples, a coverage of at least 80% of genomes GenBank Accession no. NC_006577.2 or LC315651.2 with an NGS depth of at least 3× was obtained in 158 (38%) cases. The qPCR Ct was known for 128 of these 158 samples and ranged between 10 and 32 with a mean value of 20.8 ± 4.2. These 158 obtained genomes were generated from nasopharyngeal samples collected between October 2017 and May 2022. The number of NGS reads per sample ranged between 15 028 and 10 388 629 with a mean number of 2 086 518 reads. Genome coverage ranged between 81.05% and 99.90% with a mean value of 96.09% ± 4.65%. The remaining 253 samples not included in our analyses had a genome coverage lower than 80% compared with the reference. The Ct of these 253 non‐retained samples were known for 165 samples and ranged between 20 and 36 with an average of 27.9 ± 3.7.

### 3.3 Phylogeny of HCoV‐HKU1 Genomes

**Figure 2:**
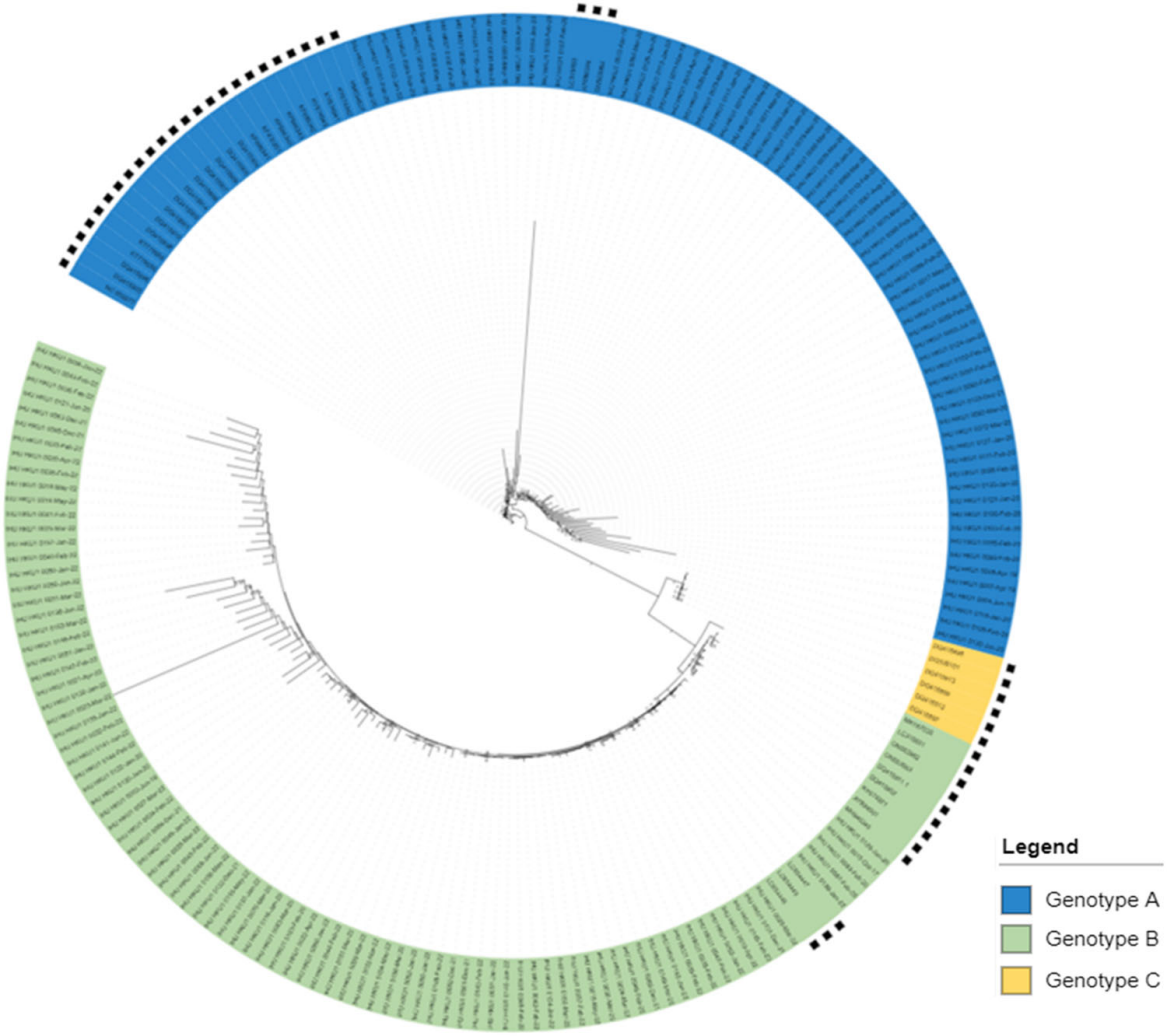
Phylogenetic tree based on HCoV‐HKU1 genomes. Asterisks indicate genomes recovered from GenBank. All other genomes were obtained in the present study.

**Figure 3:**
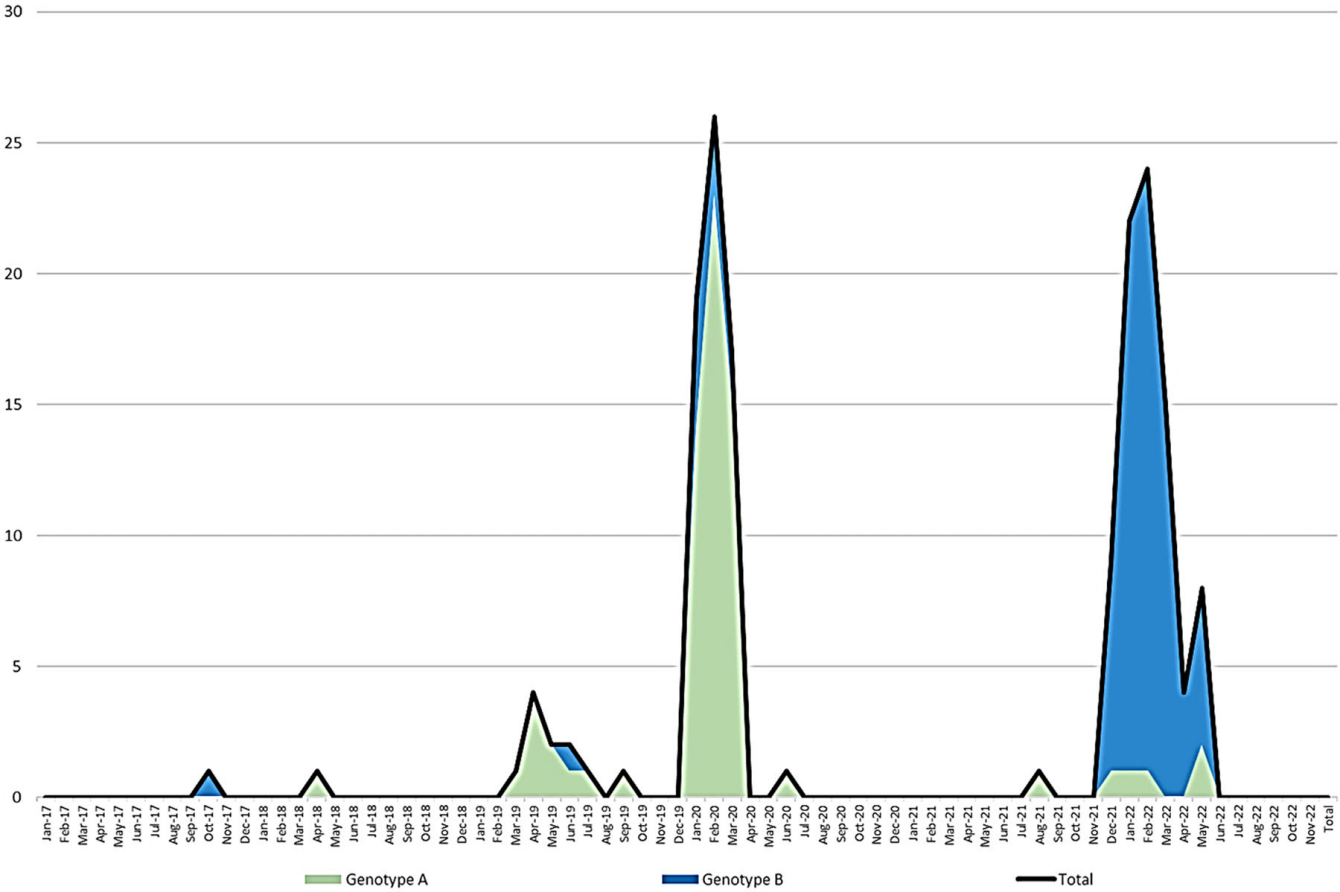
Distribution of genotypes over time for HCoV‐HKU1 genomes obtained in the present study.

Of the 158 HCoV‐HKU1 selected genomes with a coverage ≥ 80%, 69 were clustered with genotype A genomes, and 89 were clustered with genotype B genomes according to pylogeny (Figure 2). None of our sequences belonged to HCoV‐HKU1 genotype C. These results were also confirmed using the Nextclade tool [24]. Pairwise nucleotide diversity between our genotype A genomes varied between 70.0% and 99.9% with a mean of 92.2% ± 6.1%. Regarding our B genotype genomes, this diversity varied between 72.5% and 99.9% with a mean of 93.2% ± 5.8%. When we examined the distribution of viral genotypes by year, we observed that both genotypes A and B circulated at the same time, but with a predominance of one of the two genotypes during a given year. Thus, genotype A predominated in 2020 while genotype B predominated in 2022 (Figure 3).

### 3.4 HCoV‐HKU1 Mutational Patterns

#### 3.4.1 Genotype A Sequences

A total of 5686 nucleotide substitutions, 449 deletions and 848 insertions were detected in genotype A sequences obtained here compared with the reference genome NC_006577.2 dating back to 2004. To gain a better understanding of these mutations and their putative impact, we considered gene categories encompassing “structural,” “informational,” “other nonstructural” and “accessory” genes. In the informational genes category, nsp12 that encodes the RNA‐dependent RNA polymerase [33] was the gene the most affected with 339 substitutions per 1000 nucleotides compared to the reference, considering that only 22 substitutions per 1000 nucleotides were present in at least 10 genomes; and the least affected gene was nsp15 that encodes an endoribonuclease with 49 substitutions per 1000 nucleotides. Regarding structural genes, the gene encoding HE was the one the most affected by nucleotide substitutions, with 299 substitutions per 1000 nucleotides, followed by the spike gene with 183 substitutions per 1000 nucleotides, and the gene the least affected was the one encoding the envelope protein, with 12 substitutions per 1000 nucleotides. The accessory genes exhibited 57 substitutions per 1000 nucleotides (Table S2).

**Figure 4:**
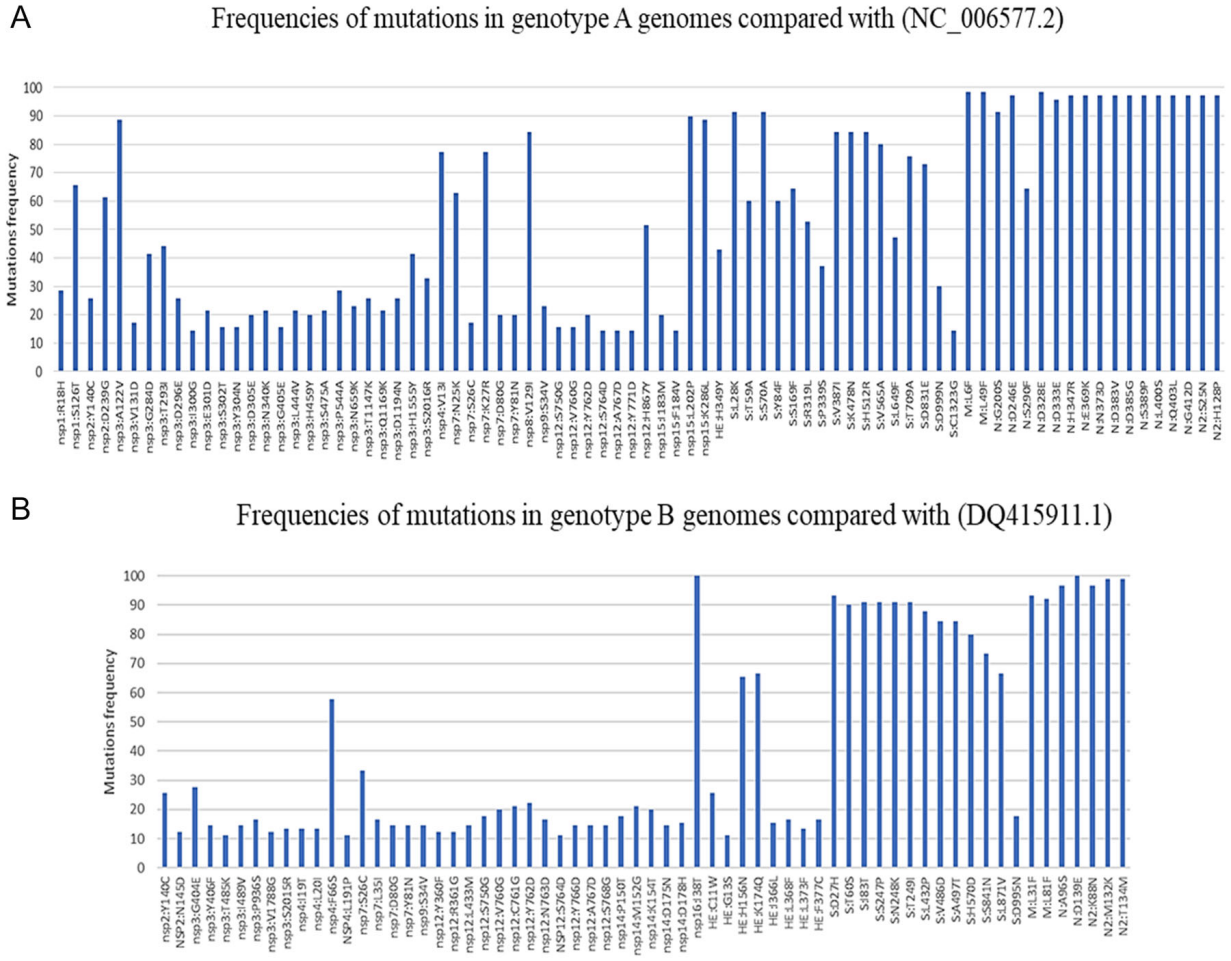
Frequencies of mutations in (A) genotype A and (B) genotype B genomes compared with references.

For these genomes belonging to genotype A, a total of 1683 amino acid substitutions were reported relatively to reference genome NC_006577.2. The number of amino acid substitutions in the genomes ranged from 58 to 635 with a mean value of 143.1 ± 93.6. The number of deletions ranged from 0 to 120 with a mean value of 22.1 ± 21.5, and the number of insertions ranged from 0 to 115 with a mean value of 8.8 ± 16.4. In the informational gene category, nsp12 had the highest diversity with 303 amino acid substitutions per 1000 amino acids, while nsp7 had none. In the structural gene category, the gene with the highest diversity was HE followed by the spike gene, with 298 and 146 substitutions per 1000 amino acids, respectively (Table S2). It is worthy to note that, of the 1683 amino acid substitutions, only 80 were present in at least 10 genomes. The genes encoding the greatest number of these 80 amino acid mutations were nsp3, which encodes a papain‐like protease (*n* = 22), the spike gene (*n* = 16), and the nucleocapsid gene (*n* = 14). Nsp5, nsp6, nsp10, nsp11, nsp13, nsp14, nsp16, E, and ORF4 genes harbored none of the amino acid substitutions present in at least 10 genomes (Figure 4A).

**Figure 5:**
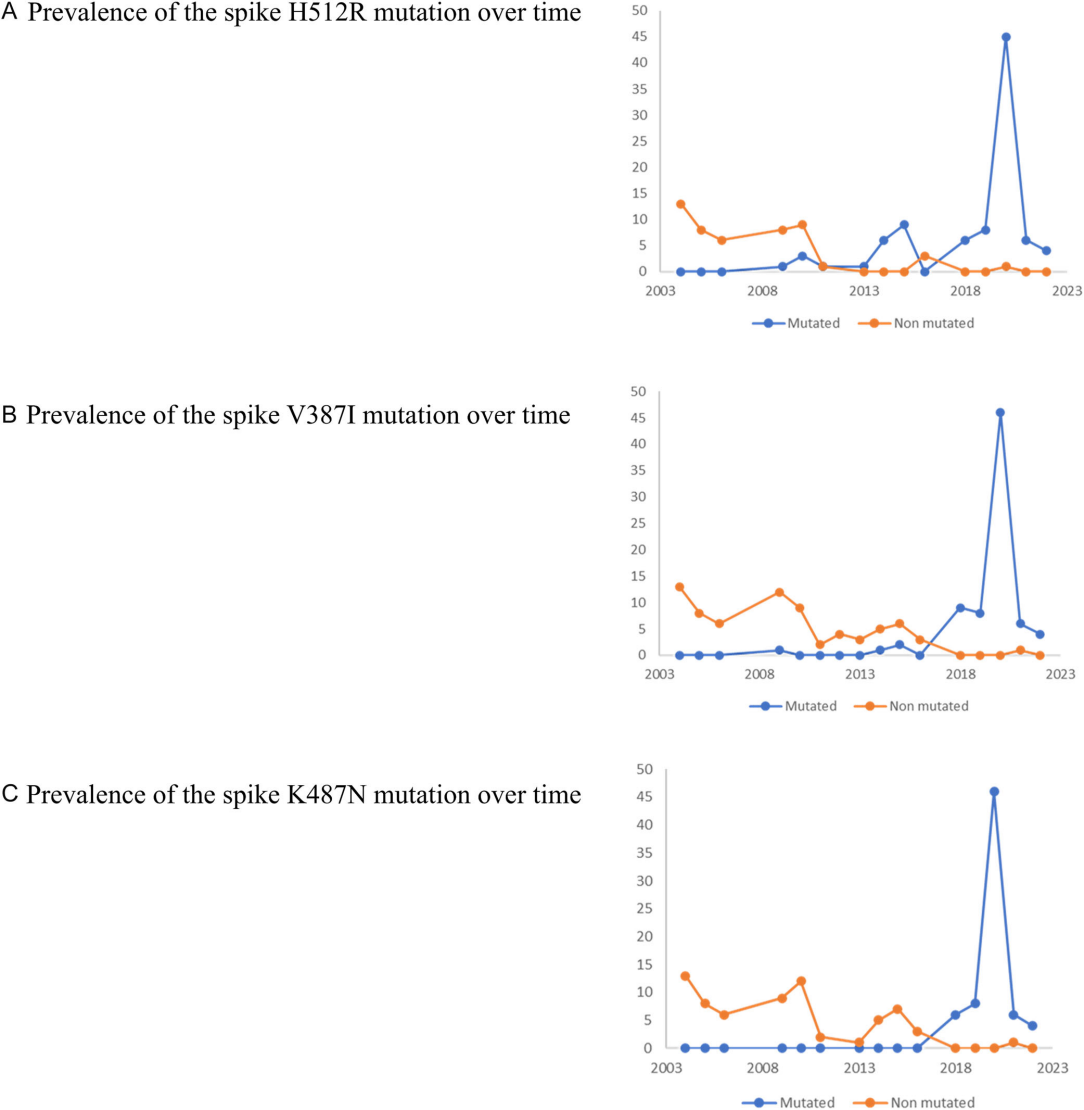
Prevalence of spike (A) H512R, (B) V387I, and (C) K487N mutations over time.

The H512R mutation observed relative to the 2004 reference genome (NC_006577.2) in the spike gene of genotype A genomes obtained here was reported to be involved in antibody neutralization [34, 35]. All HCoV‐HKU1 genotype A spikes covering this position were retrieved from Genbank and analyzed with those obtained here. According to GenBank data, the H512R mutation appeared in 2009 and became the majority mutation among genomes over time (Figure 5A). For year 2009, only one sequence (11%) was detected as carrying this mutation. Between 2010 and 2016, 20/33 (60%) of the sequences carried this mutation. No genotype A sequences were detected for year 2017 either in Genbank or in the present study. For the years 2018 and 2019, all 14 sequences analyzed in both GenBank and the present study harbored this mutation. For the year 2020, only one sequence was found in GenBank and it did not carry this mutation, whereas all 45 sequences from the present study carried this mutation. Finally, for 2021 and 2022, all 10 sequences obtained here carried the mutation (Table 1). The first sequence carrying the H512R mutation detected in 2009 originated from the United States while, overall, genomes deposited in GenBank and carrying this mutation originated from the USA, Thailand, China, Japan, and Russia; the present study further shows the presence of this mutation in genotype A sequences circulating in the south of France.

**Table 1:** Proportions of spike H512R, V387I, and K487N mutations over time.

		Year										
		< 2009	2009	2010–2016	2017	2018	2019	2020	2021	2022	2023	Any year
Sequences harboring the mutation/covering the position (N [%])												
H512R												
Present study		—	—	—	—	1/1 (100%)	7/7 (100%)	45/45 (100%)	2/2 (100%)	3/3 (100%)	—	58/58 (100%)
GenBank		0/27 (0%)	1/9 (11%)	20/33 (60%)	—	5/5 (100%)	1/1 (100%)	0/1 (0%)	4/4 (100%)	1/1 (100%)	—	32/81 (39%)
Total		0 (0%)	1 (11%)	20 (60%)	—	6 (100%)	8 (100%)	45 (100%)	6 (100%)	4 (100%)	—	90/139 (64%)
V387I												
Present study		—	—	—	—	1/1 (100%)	7/7 (100%)	45/45 (100%)	2/2 (100%)	3/3 (100%)	—	58/58 (100%)
GenBank		0/27 (0%)	1/13 (7%)	3/35 (8%)	—	8/8 (100%)	1/1 (100%)	1/1 (100%)	4/5 (80%)	1/1 (100%)	—	19/91 (21%)
Total		0 (0%)	1 (7%)	3 (8%)	—	9 (100%)	8 (100%)	46 (100%)	6 (85%)	4 (100%)	—	77 (51%)
K478N												
Present study		—	—	—	—	1/1 (100%)	7/7 (100%)	45/45 (100%)	2/2 (100%)	3/3 (100%)	—	58 (100%)
GenBank		0/27 (0%)	0/9 (0%)	0/30 (0%)	—	5/5 (100%)	1/1 (100%)	1/1 (100%)	4/5 (80%)	1/1 (100%)	—	12/79 (15%)
Total		0 (0%)	0 (0%)	0 (0%)	—	6 (100%)	8 (100%)	46 (100%)	6 (85%)	4 (100%)	—	70/137 (51%)
H512R + V387I												
Present study		—	—	—	—	1/1 (100%)	7/7 (100%)	45/45 (100%)	2/2 (100%)	3/3 (100%)	—	58 (100%)
GenBank		0/27 (0%)	1/9 (11%)	3/29 (10%)	—	5/5 (100%)	1/1 (100%)	0/1 (0%)	4/4 (100%)	1/1 (100%)	—	15 (19%)
Total		0 (0%)	1 (11%)	3 (10%)	—	6 (100%)	8 (100%)	45 (100%)	6 (100%)	4 (100%)	—	73/135 (54%)
H512R + K478N												
Present study		—	—	—	—	1/1 (100%)	7/7 (100%)	45/45 (100%)	2/2 (100%)	3/3 (100%)	—	58 (100%)
GenBank		0/27 (0%)	0/9 (0%)	0/30 (0%)	—	5/5 100%)	1/1 (100%)	0/1 (0%)	4/4 (100%)	1/1 (100%)	—	11/78 (14%)
Total		0 (0%)	0 (0%)	0 (0%)	—	6 (100%)	8 (100%)	45 (100%)	6 (100%)	4 (100%)	—	69/136 (50%)
H512R + V387I + K478N												
Present study		—	—	—	—	1/1 (100%)	7/7 (100%)	45/45 (100%)	2/2 (100%)	3/3 (100%)	—	58 (100%)
GenBank		0/27 (0%)	0/9 (0%)	0/27 (0%)	—	5/5 (100%)	1/1 (100%)	0/1 (0%)	4/4 (100%)	1/1 (100%)	—	11/75 (14%)
Total		0 (0%)	0 (0%)	0 (0%)	—	6 (100%)	8 (100%)	45 (100%)	6 (100%)	4 (100%)	—	69/133 (52%)
V387I + K478N												
Present study		—	—	—	—	1/1 (100%)	7/7 (100%)	45/45 (100%)	2/2 (100%)	3/3 (100%)	—	58 (100%)
GenBank		0/27 (0%)	0/9 (0%)	0/27 (0%)	—	5/5 (100%)	1/1 (100%)	1/1 (100%)	4/5 (80%)	1/1 (100%)	—	12/76 (15%)
Total		0 (0%)	0 (0%)	0 (0%)	—	6 (100%)	8 (100%)	46 (100%)	6 (85%)	4 (100%)	—	70/134 (52%)

Another amino acid mutation in the spike, V387I, was observed in the sequences obtained here. Hence, we collected from GenBank all HCoV‐HKU1 spike sequences that covered this amino acid position. We observed that this mutation appeared in 2009, being present in a single sequence out of 13 from GenBank (Figure 5B). Thereafter, between 2010 and 2016, this mutation was observed in 3 (8%) of 38 sequences from GenBank. For years 2018, 2019, and 2020, all 63 sequences (from GenBank and the present study) carried this mutation. For 2021, the two sequences from the present study and four of the five sequences from GenBank carried this mutation. Finally, for 2022, the four sequences from GenBank and from the present study all carried this mutation (Table 1). Regarding its geographical distribution, this mutation was observed for the first time in the USA, then was also observed in China, Japan, and Russia, and in France as shown in the present study. K478N was also present in the spike of genotype A sequences obtained here. It was first detected in sequences available from GenBank in 2018 and continued to be detected since then (Figure 5C). All 64 sequences obtained for years 2018, 2019, 2020, and 2022 carried this mutation, and it was the case for 2021 for four of the five sequences available from GenBank and the two genotype A sequences from the present study (Table 1). Overall, this mutation was observed in China, Japan, the USA, and France.

We then looked at whether the H512R mutation was linked to V387I and/or K478N mutations. We first determined the proportion of sequences with the H512R and V387I mutations (Table 1). In 2009, the only sequence with the H512R mutation also carried V387I. For sequences obtained from samples collected between 2010 and 2016, only three of 29 sequences carried these two mutations combined. Then, all 69 sequences between 2018 and 2022 available from GenBank or from the present study carried these two mutations combined. Regarding mutations H512R and K478N, all 69 sequences carried these two mutations combined (Table 1). Regarding mutations V387I and K478N, they were combined in all 69 sequences except in 1 of the 5 genomes available from GenBank for 2021; but unfortunately, these latter genomes did not cover spike amino acid 512 (Table 1). Finally, to investigate further how these three mutations (H512R, V387I, and K478N) were linked, we recovered all the sequences that covered their positions, and determined that all 69 such sequences harbored these three mutations combined (Table 1). Additional comparative genomics results can be found in Supporting Information S1: Results.

#### 3.4.2 Genotype B Sequences

For the HCoV‐HKU1 genotype B genomes, 6129 nucleotide substitutions, 428 deletions, and 801 insertions relative to the reference genome DQ415911.1 dating back to 2005 were observed. Among informational genes, the gene with the greatest nucleotide diversity was nsp12 with 290 substitutions per 1000 nucleotides. In the nonstructural gene category, the nsp9 gene showed the greatest diversity (424 substitutions per 1000 nucleotides) and nsp1 showed the lowest diversity (54 substitutions per 1000 nucleotides). Among structural genes, the HE gene showed the greatest diversity with 475 substitutions per 1000 nucleotides, followed by the spike gene with 211 substitutions per 1000 nucleotides. Accessory genes exhibited 160 substitutions per 1000 nucleotides overall (Table S4).

Compared with this reference genome DQ415911.1, 1802 amino acid substitutions were detected within genome sequences obtained here. The number of amino acid substitutions ranged from 22 to 245 with a mean value of 60.3 ± 44.4; the number of deletions ranged from 0 to 46 with a mean value of 8.3 ± 9.6; and the number of insertions ranged from 0 to 36 with an average of 2.8 ± 5.3. Among informational proteins, nsp14 was that with the greatest number of substitutions per 1000 amino acids (*n* = 305) and nsp15 was that with the lowest number of mutations (*n* = 117). Among structural proteins, HE showed the greatest diversity followed by the envelope, with 382 and 292 substitutions per 1000 amino acids, respectively. The spike harbored 103 substitutions per 1000 amino acids. The accessory protein coding for ORF4 exhibited 183 substitutions per 1000 amino acids (Table S4). However, of all the 1802 amino acid substitutions, only 64 were found in at least 10 genomes. Proteins with no amino acid substitution in at least 10 genomes were nsp1, nsp5, nsp6, nsp8, nsp10, nsp11, nsp13, nsp15, E, and ORF4 (Figure 4B). Additional comparative genomics results can be found in Supporting Information S1: Results.

### 3.5 Molecular Modeling of HCoV‐HKU1 Spikes

**Figure 6:**
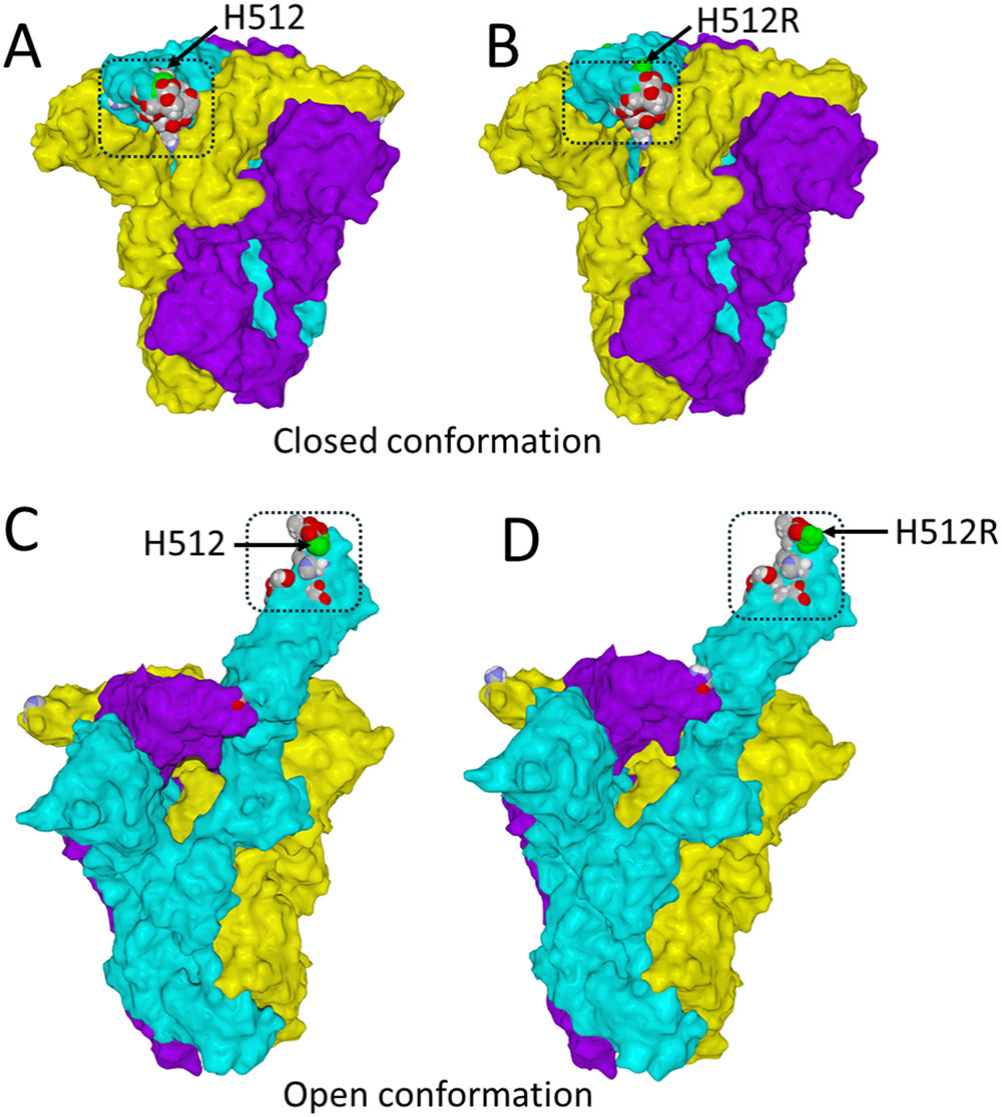
Location of H512R mutation in the trimeric spike of HCo‐V‐HKU1 in the closed and open conformation. (A) Wild‐type trimer with H512 (green atomic spheres) in the closed conformation. The three subunits are colored in cyan (chain A), yellow (chain B), and purple (chain C). Amino acid residues belonging to the receptor binding motif (RBM, rectangle in dotted lines) are shown in atomic spheres (carbon in green, oxygen in red, nitrogen in blue, and hydrogen in gray). (B) H512R mutant in the closed conformation, same legend as in panel A. (C) Wild type trimer in the open conformation. (D) H512R mutant in the open conformation. Note that in panels C and D, the RBM is totally accessible to TMPRSS2. The structures of the wild‐type HCoV ‐‐HKU1 trimer in the closed and open conformations were retrieved from pdb 8ohn and 8opn, respectively. Mutation H512R was introduced in these files and submitted to molecular modeling.

We then carried out an in silico molecular modeling of the HCoV‐HKU1‐spike protein with the H512R mutation and the two other mutations, V387I and K478N, observed in genotype A genomes obtained here. In the closed state, the receptor binding motif (RBM) of the spike protein is initially partially masked and definitely not accessible to the TMPRSS2 receptor (Figure 6A). It has been assigned to a region globally encompassing amino acids 505–532, with the addition of residues 488 and 554 [36]. The H512R mutation is close to critical amino acids that control binding to the receptor, such as H488 and W515 (Figure 6B). Since H512 is exposed on the surface of the spike protein, its substitution by a cationic amino acid (arginine) is perfectly tolerated by the protein trimer. Indeed, there is very little difference between the 3D structures of wild‐type and H512R mutant trimers in the closed conformation. Once bound to sialic acids, the trimer undergoes a dramatic conformational rearrangement that propels the receptor binding domain (RBD) toward the TMPRSS2 receptor (Figure 6C). As expected, both the wild‐type H512 (Figure 6C) and mutant H512R (Figure 6D) are accessible for the receptor in the open conformation of the trimeric spike.

**Figure 7:**
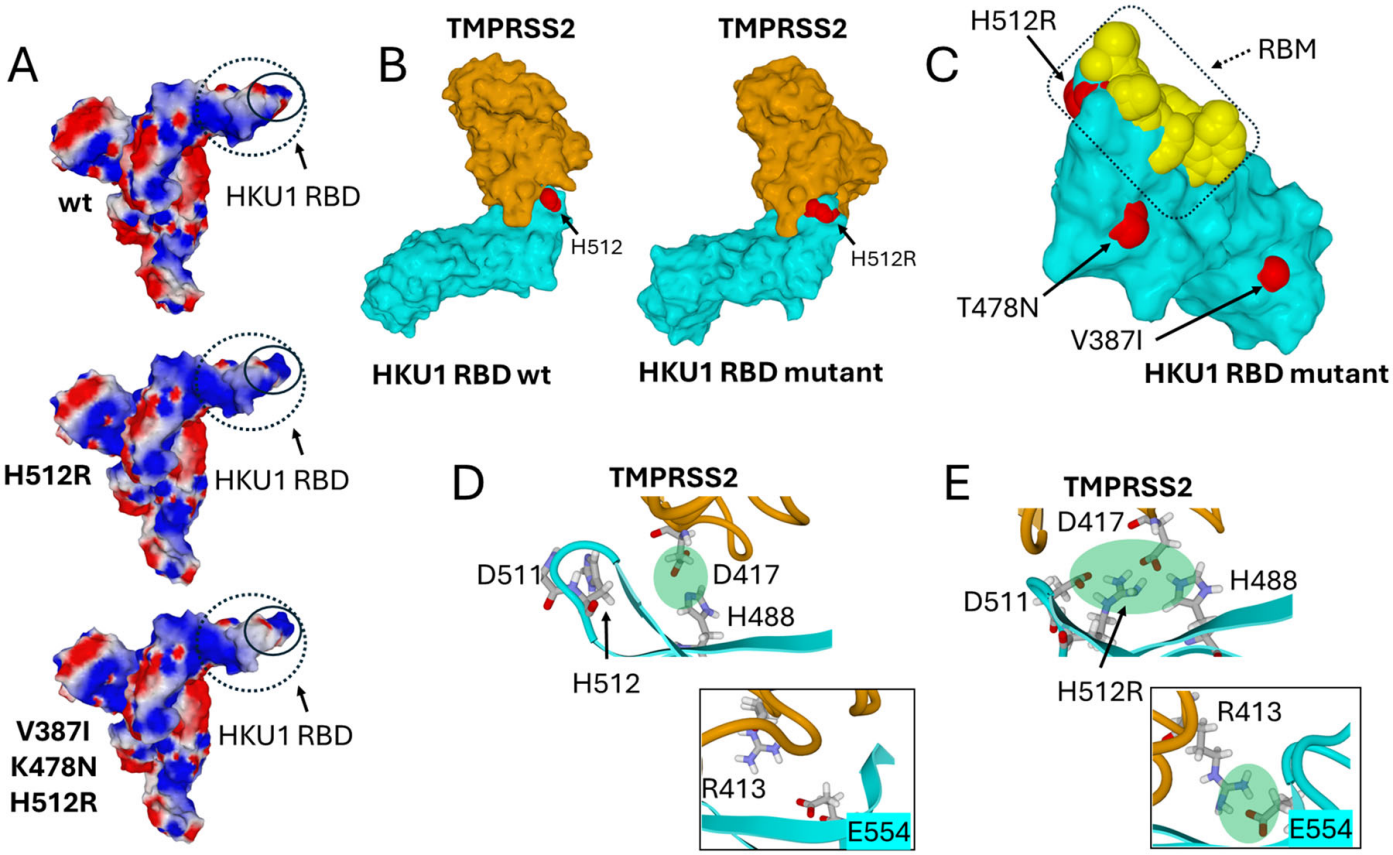
Impact of mutations H512R, K478N, and V387I on HCoV‐HKU1 spike in the open conformation. (A) Electrostatic surface potential of wild‐type, single mutant H512R and triple mutant H512R, K478N, and V387I calculated in spike monomers. These three mutations are all located within the receptor binding domain (RBD, dotted circle). Electronegative areas are in red, electropositive in blue and neutral in white. Note the significant increase of electropositive surfaces in the RBM (solid circle) of the single and triple mutant spikes. (B) TMPRSS2 bound to wild‐type HCoV‐HKU1 RBM (left panel) or triple mutant (right panel). TMPRSS2 is colored in orange, the HCoV‐HKU1 RBD in cyan. Amino acids H512 (wild‐type) and H512R (mutant) are represented in red atomic spheres. (C) Focus on the amino acid residues that constitute the RBM of HCoV‐HKU1 (dotted rectangle, amino acids in atomic spheres) in the RBD (colored in cyan). Mutant amino acids are represented in red atomic spheres. Note that only H512R is in close contact with the RBM; T478N and V387I are not directly linked to the RBM. (D) TMPRSS2 (orange ribbons) bound to wild‐type HCoV‐HKU1 RBM (cyan ribbons). Note that H488 (HCoV‐HKU1) interacts with D417 (TMPRSS2). The binding zone is indicated by a green disk. Inset: lack of interaction between E554 (HCoV‐HKU1) and R413 (TMPRSS2). The atomic coordinates were retrieved from pdb 8vgt. (E) Molecular dynamics simulations (MDS) of TMPRSS2 binding to the triple mutant of HCoV‐HKU1 (open conformation). Inset: during the binding process, an electrostatic bond is established between E554 (HCoV‐HKU1) and R413 (TMPRSS2). H512R is predicted to form a high‐energy complex (green disk) involving D511 and H488 (HCoV‐HKU1) and D417 (TMPRSS2). This minimizes the initial electrostatic repulsion between D511 and D417.

The H512R mutation has a profound impact on the electrostatic surface potential of the RBM (Figure 7A). Compared with the wild‐type trimer, the H512R mutant is characterized by an increased electropositive area at the tip of the RBM. This increase in electropositivity is then partially compensed by secondary mutations V387I and K478N, but not at the tip of the RBD whose electrostatic surface potential remains highly positive. We used molecular modeling approaches to predict the impact of these mutations on the TMPRSS2‐HCo‐V‐HKU1 complex (Figure 7B–E). Among the three mutations, only H512R appeared to be involved in the formation of this complex (Figure 7B,C), since both K478N and V387I were clearly outside the interaction site. These observations confirmed that K478N and V387I are secondary mutations that finely tune the architecture of the RBM already bearing H512R.

During the binding reaction, we detected the formation of an electrostatic bond between the gamma‐carboxylate group of E505 (HCoV‐HKU1 RBM) and the cationic guanidinium group of R470 (TMPRSS2), in full agreement with recently published cryoelectromicroscopy data [36]. We also observed a similar electrostatic bond between the beta‐carboxylate group of D511 and the cationic group of H512R (Figure 7D,E). This intramolecular bond prevents the electrostatic repulsion between D417 (TMPRSS2) and D511 (HCoV‐HKU1 RBM) and stabilizes the underneath loop displaying H488 (see comparison of Figures 7D,E). Since H488 interacts with D417 in the TMPRSS2‐HCoV‐HKU1 complex [36], a conformational adjustment is required to bring both amino acids into close contact. Indeed, the cationic group of H512R initiates a rearrangement of the binding interface, resulting in the formation of a network of electrostatic interactions involving H512R, D511, H488 (HCoV‐HKU1), and D417 (TMPRSS2). This is predicted to induce a slight conformational change that allows the formation of an electrostatic interaction between E554 (HCoV‐HKU1) and R413 (TMPRSS2).

**Figure 8:**
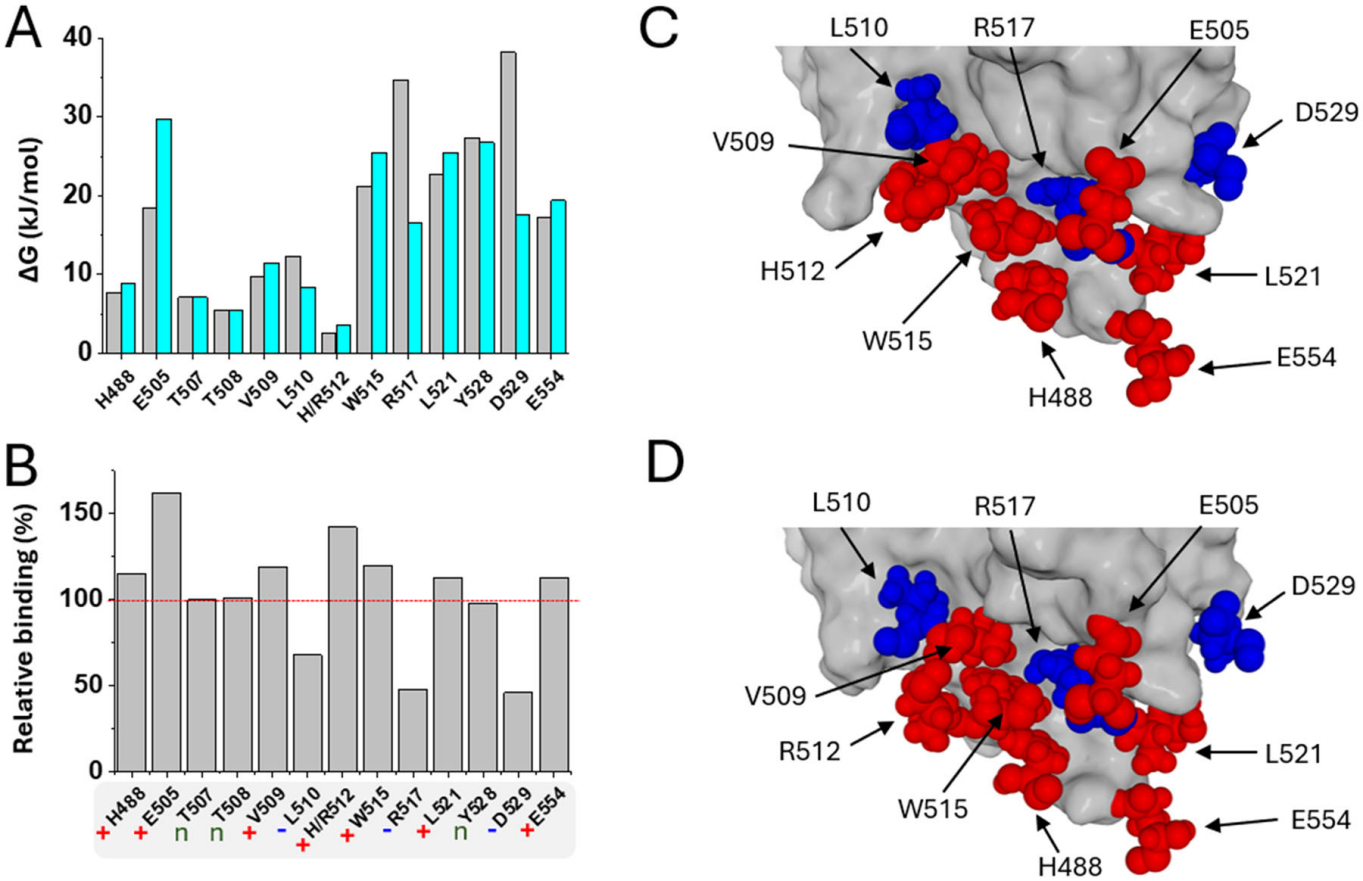
Remodeling of the TMPRSS2 binding site induced by mutations H512R, K478N, and V387I in HCoV‐HKU1 spike. (A) Calculations of the energy of interaction amino acid by amino acid. Only the amino acid residues that significantly contribute to the HCoV‐HKU1‐TMRSS2 (Δ*G* > 5 kJ*·*mol^−^^1^) are considered, (except for mutation H512R). Gray histograms, wild‐type HCoV‐HKU1 spike; cyan histograms, triple mutant HCoV‐HKU1 spike. The data were obtained from the Ligand Energy Inspector function of the Molegro software. (B) Relative contribution of each amino acid residue with the (TMPRSS2‐wild type HCoV‐HKU1 spike) complex taken as 100%. Amino acids that have an increased energy of interaction in the triple mutant HCoV‐HKU1 spike are note by a red “plus” symbol, those with decreased energy of interaction are noted by a blue “minus” symbol, and those unaffected by the mutations are noted by a green “n” (for neutral). The red dashed line indicates 100% (no effect on the energy of interaction). (C) TMPRSS2 binding site for the wild‐type HCoV‐HKU1 spike protein. Amino acids listed in the histograms of panel B are shown in atomic spheres with a size reduced by 0.5 for clarity. The TMPRSS2 surface is colored gray. (D) TMPRSS2 binding site for the triple mutant HCoV‐HKU1 spike protein. Note the rearrangements of the TMPRSS2 surface revealed by molecular dynamics simulations.

A complete RBM study of the HCoV‐HKU1 spike allowed us to analyze in detail the contribution of each amino acid in the interaction with TMPRSS2 (Figure 8A). For the wild‐type spike protein, amino acids the most strongly involved are E505, W515, R517, L521, Y528, D529, and E554. The H512 mutation site contributes to the complex but to a lesser extent. For the triple mutant spike protein, the same amino acids stabilize the complex, but with notable differences in their energy of interaction (Figure 8A). Some of these amino acids interact more strongly with the RBM of the triple mutant, others more weakly while still for others there is no notable difference (Figure 8A,B). The most significant increases regard E505 and the mutation site H512R. The most important decreases regard residues R517 and D529. Finally, T507, T508, and Y528 are not affected; their energy of interaction is similar for the wild form and the triple mutant. These results suggest that the TMPRSS2 binding site on the HCoV‐HKU1 spike protein is generally conserved in the triple mutant. However, there are significant variations in the energy of interaction of certain amino acids, either upward or downward. These adjustments are illustrated in the structural models of Figure 8C (wild‐type spike protein) and 8D (triple mutant). MDS show the reorientations of the amino acids of the spike protein but also the conformational modulations of the TMPRSS2 protein which definitely contribute to the fine‐tuning of the complex. This is particularly clear in the left part of the TMPRSS2 structure that must adapt to the H512R substitution. In these models, the amino acids for which interaction energy is increased in the triple mutant are colored in red, those for which interaction energy is decreased are in blue, to use the same color code as for Figure 8B. Overall, due to these complex rearrangements, the H512R mutation is predicted to affect both the kinetics and the affinity of the TMPRSS2‐HCoV‐HKU1 complex, explaining the selection of this mutant over the wild‐type virus.

## 4 Discussion

Coronaviruses can cross the species barrier into humans from other animals, making them candidates for emergence events, as observed with the recent SARS‐CoV‐2 pandemic [1, 37, 38]. Here we analyzed 158 genomes of HCoV‐HKU1, which is one of the four coronaviruses that were endemic before the SARS‐CoV‐2 emergence. This set of genomes represents about 1.5 times the number of genomes previously available for this virus in the GenBank database, which was, and remains a dramatically small set in comparison with more than 15 million SARS‐CoV‐2 genomes available. In addition, in GenBank, most HCoV‐HKU1 genomes were from samples originating from the USA and China, and for the 2017–2022 period, only 55 HCoV‐HKU1 genomes were available in GenBank. Recently, Ye et al. and Mah et al. reported each four HCoV‐HKU1 genomes recovered from respiratory samples collected between 2016 and 2018 in China and between 2010 and 2016 in Singapore or Tanzania, respectively [39, 40]. These genomes were obtained by metatranscriptomics after enrichment through nucleic acid purification and after culture isolation, respectively. Moreover, a single genome was available from France, which dates back to 2005. Thus, our study carried out on nasopharyngeal samples collected between 2017 and 2022 substantially expands the number of genomes available for this virus at the global scale and is the one that reports the largest number of genomic sequences.

Most of the viral genomes described here were obtained from samples collected in 2020 and 2022. This can be explained by the SARS‐CoV‐2 pandemic that led to test massively people with symptoms of respiratory viruses. Most of the respiratory samples from which genomes were obtained were collected between January and March months, with peaks between January and February, which is in line with studies showing that in temperate countries HCoV‐HKU1 incidence peaks occur during these two latter months [11, 12, 13]. No infection with HCoV‐HKU1 was diagnosed in our clinical microbiology laboratory between July 2020 and June 2021, which may be explained in part by the public health measures implemented during this period such as confinement and mask‐wearing that might have resulted in a reduction of cases, as previously reported in our area [41].

We identified HCoV‐HKU1 of genotypes A and B. Co‐circulation of these two genotypes has been reported in many studies [5, 8, 9, 42, 43]. Even if there was a co‐circulation of the two genotypes every year in previous studies and the present study, there was a predominance of one genotype over the other according to the year. Besides, no genotype C has been detected since 2005 [43]. Regarding nucleotide substitutions, whatever the genotype studied, at the level of informational genes, the Nsp12 gene that encodes the RNA‐dependent RNA polymerase is the one that exhibited the greatest nucleotide diversity, which was not reported to be the case for SARS‐CoV‐2 in a previous study [25]. Nevertheless, most of the nucleotide mutations detected in Nsp12 were only found in a few genomes. In the category of structural genes, the envelope‐encoding gene showed the lowest diversity among genotype A genomes. In a previous study that compared genomes of genotypes A to those of genotypes B and C, the genes exhibiting the greatest diversity were those encoding the envelope, the spike, and the HE, while the other genes were relatively conserved [43]. The present data are consistent with those of previous studies showing that the spike protein is encoded by the most variable gene within this virus [44]. For genotype A sequences, some amino acid substitutions (L28K, S70A, V387I, K478N) in the spike we observed here were previously reported [43] and are located in the S1 subunit. Besides, spike amino acids V509, L510, D511, H512, W515, or R517 were reported to have an impact on the sensibility of immune responses based on structural prediction [34].

In the present study, at one of these positions, amino acid substitution H512R was detected, which was previously reported [35]. After analyzing all the HCoV‐HKU1 spike sequences available in GenBank as well as the sequences obtained here, it appeared that this mutation was present for the first time in early 2009, and that 512R thereafter become over time the majority amino acid, and almost the only one in circulating genotype A genomes. This H512R mutation has been detected in the USA, China, Thailand, Japan, and France as shown here. This mutation was first observed in association with another mutation, V387I, but we determined that these two mutations were not always associated. Mutation K487N was present in genomes obtained from more recent samples, in 2018. Then, since 2018, the majority of genotype A sequences harbored these three mutations combined. The present study points out that H512R can occur alone but tends to be accompanied by mutations V387I and K478N. Although, the low number of sequences available in GenBank and through our study may lead to an inaccurate representation of the global HCoV‐HKU1 genome diversity, we may consider H512R, and to a lesser extent mutations V387I and K478N, as “hyperfertile,” similar to a previous study conducted on SARS‐CoV‐2 [25].

Structural studies of the three mutations H512R, V387I, and K478N revealed new information on the evolution of the HKU1 coronavirus. H512R, is well tolerated by the trimeric spike. The appearance of a cationic charge located at the tip of the RBM increases the electrostatic surface potential of the trimeric spike. This effect has been previously observed with other RNA viruses such as HIV‐1 and SARS‐CoV‐2, which use lipid rafts as primary adhesion sites on host cells [45]. The recent demonstration that sialic acid binding triggers HKU1 spike opening [46] confirmed our model of a dual glycolipid‐protein receptor for virus entry mechanisms [47]. Since lipid rafts are enriched in gangliosides, which contain sialic acids, they are negatively charged and attract more rapidly mutant virus with increased electropositivity. This effect explains how the evolution of those viruses is driven by host cell membranes [48]. However, compensatory mechanisms often occur to balance the global electrostatic surface potential of mutant viruses, as remarkably shown for the Omicron lineage of SARS‐CoV‐2. In this case, the electrostatic potential gradually increases in the RBD and simultaneously decreases in the N‐terminal domain of the spike protein [48]. Yet, the compensation does not result in a return to the initial state since, as the variants come one after the other, the electrostatic potential becomes stronger and stronger, as observed from Alpha to Delta SARS‐CoV‐2 variants [27] and then in the Omicron lineage [48]. In this respect, it is likely that V387I and K478N, which appeared after the selection of H512R, are compensatory mutations that finely tune the electrostatic surface potential of the trimeric spike. The second important impact of H512R concerns the binding of TMPRSS2. MDS confirmed the global geometry of the TMPRSS2‐HKU1 complex and gave some insights on the potential effects of the H512R mutation. We propose that H512R forms an intramolecular electrostatic bond with D511, which minimizes the electrostatic repulsion between D511 (HKU1) and D417 (TMPRSS2) (Figure 7D,E). This mechanism is expected to improve the initial binding steps between the spike trimer and the TMPRSS2 receptor, which may then involve subtle conformational rearrangements. This mechanism (i) facilitates the interaction between H488 (HKU1) and D417 (TMPRSS2) and (ii) reinforces the complex through an electrostatic network of interactions involving H512R with D417. Overall, our molecular modeling studies suggest that H512R can significantly improve TMPRSS2 binding to mutant viruses (Figure 8), through both kinetics and affinity parameters.

The present study is not necessarily representative of what is happening worldwide. It was carried out for a given location and a given period of time, and the number of data available worldwide has been revealed here to be limited. Notwithstanding, it contributes to our understanding of the evolution of HCoV‐HKU1 and also considerably enrich the database of HCoV‐HKU1 genomes. We show here that genomic monitoring directly from respiratory samples combined with protein structural predictions analysis can detect and explore the emergence of HCoV‐HKU1 lineages. Further work on a large number of samples would be necessary to be able estimating whether or not the different HCoV‐HKU1 genotypes and lineages may be associated with particular clinical presentations. Also, future studies conducted in our institute or by other teams should isolate several HCoV‐HKU1 strains of various lineages for their phenotypic characterization.

## Author Contributions

Conceived and designed the experiments: Bernard La Scola and Philippe Colson. Contributed materials and analysis tools: Houmadi Hikmat, Lorlane Le Targa, Céline Boschi, Justine Py, Aurélie Morand, Jean‐Christophe Lagier, Sarah Aherfi, Jacques Fantini, Bernard La Scola, and Philippe Colson. Analyzed the data: Houmadi Hikmat, Céline Boschi, Jacques Fantini, Bernard La Scola, and Philippe Colson. Writing—original draft preparation: Houmadi Hikmat, Bernard La Scola, Jacques Fantini, Bernard La Scola, and Philippe Colson. Writing—review and editing: All authors. All authors have read and agreed to the published version of the manuscript.

## Ethics Statement

The present study has been registered on the Health Data Access Portal of Marseille public and university hospitals (Assistance Publique‐Hôpitaux de Marseille [AP‐HM]) with no. PADS24‐190 and was approved by the Ethics and Scientific Committee of AP‐HM.

## Conflicts of Interest

Lorlane Le Targa works for Biosellal, a company located in Dardilly, France. The other authors declare no conflicts of interest.

## Supporting information

Supporting information.

## Data Availability

HCoV‐HKU1 genomes analyzed here have been submitted to the GenBank sequence database [14] (https://www.ncbi.nlm.nih.gov/genbank/) (GenBank Accession no: PP356751 ‐ PP356908).

## References

[1] V. M. Corman , D. Muth , D. Niemeyer , and C. Drosten , “Hosts and Sources of Endemic Human Coronaviruses,” Advances in Virus Research 100 (2018): 163–188, 10.1016/bs.aivir.2018.01.001. 29551135 PMC7112090

[2] F. Kakuya , R. Terao , H. Onoda , et al., “Epidemiology of Endemic Human Coronavirus Infection During the COVID‐19 Pandemic,” Journal of Infection and Chemotherapy 30, no. 5 (2024): 400–405, 10.1016/j.jiac.2023.11.012. 37979777

[3] J. R. Otieno , J. L. Cherry , D. J. Spiro , M. I. Nelson , and N. S. Trovão , “Origins and Evolution of Seasonal Human Coronaviruses,” Viruses 14, no. 7 (2022): 1551, 10.3390/v14071551. 35891531 PMC9320361

[4] P. C. Y. Woo , S. K. P. Lau , C. Chu , et al., “Characterization and Complete Genome Sequence of a Novel Coronavirus, Coronavirus HKU1, From Patients With Pneumonia,” Journal of Virology 79, no. 2 (2005): 884–895, 10.1128/JVI.79.2.884-895.2005. 15613317 PMC538593

[5] P. C. Y. Woo , S. K. P. Lau , C. C. Y. Yip , et al., “Comparative Analysis of 22 Coronavirus HKU1 Genomes Reveals a Novel Genotype and Evidence of Natural Recombination in Coronavirus HKU1,” Journal of Virology 80, no. 14 (2006): 7136–7145, 10.1128/JVI.00509-06. 16809319 PMC1489027

[6] R. J. G. Hulswit , Y. Lang , M. J. G. Bakkers , et al., “Human Coronaviruses OC43 and HKU1 Bind to 9‐O‐Acetylated Sialic Acids via a Conserved Receptor‐Binding Site in Spike Protein Domain A,” Proceedings of the National Academy of Sciences 116, no. 7 (2019): 2681–2690, 10.1073/pnas.1809667116. PMC6377473 30679277

[7] N. Saunders , I. Fernandez , C. Planchais , et al., “TMPRSS2 Is a Functional Receptor for Human Coronavirus HKU1,” Nature 624, no. 7990 (2023): 207–214, 10.1038/s41586-023-06761-7. 37879362 PMC11331971

[8] Y. Jin , J. R. Song , Z. P. Xie , et al., “Prevalence and Clinical Characteristics of Human CoV‐HKU1 in Children With Acute Respiratory Tract Infections in China,” Journal of Clinical Virology 49, no. 2 (2010): 126–130, 10.1016/j.jcv.2010.07.002. 20702134 PMC7108232

[9] G. Gerna , E. Percivalle , A. Sarasini , et al., “Human Respiratory Coronavirus HKU1 Versus Other Coronavirus Infections in Italian Hospitalised Patients,” Journal of Clinical Virology 38, no. 3 (2007): 244–250, 10.1016/j.jcv.2006.12.008. 17222582 PMC7108341

[10] S. Su , G. Wong , W. Shi , et al., “Epidemiology, Genetic Recombination, and Pathogenesis of Coronaviruses,” Trends in Microbiology 24, no. 6 (2016): 490–502, 10.1016/j.tim.2016.03.003. 27012512 PMC7125511

[11] P. Liu , L. Shi , W. Zhang , et al., “Prevalence and Genetic Diversity Analysis of Human Coronaviruses Among Cross‐Border Children,” Virology Journal 14 (2017): 230, 10.1186/s12985-017-0896-0. 29166910 PMC5700739

[12] E. R. Gaunt , A. Hardie , E. C. J. Claas , P. Simmonds , and K. E. Templeton , “Epidemiology and Clinical Presentations of the Four Human Coronaviruses 229E, HKU1, NL63, and OC43 Detected Over 3 Years Using a Novel Multiplex Real‐Time PCR Method,” Journal of Clinical Microbiology 48, no. 8 (2010): 2940–2947, 10.1128/JCM.00636-10. 20554810 PMC2916580

[13] L. J. Cui , Z. Chen , Z. Ting , R. J. Lu , and X. Zd . Human Coronaviruses HCoV‐NL63 and HCoV‐HKU1 in Hospitalized Children With Acute Respiratory Infections in Beijing, China. 2011, https://www.hindawi.com/journals/av/2011/129134/. 10.1155/2011/129134 PMC3265292 22315599

[14] E. W. Sayers , M. Cavanaugh , K. Clark , et al., “GenBank 2023 Update,” Nucleic Acids Research 51, no. D1 (2023): D141–D144, 10.1093/nar/gkac1012. 36350640 PMC9825519

[15] S. Khare , C. Gurry , L. Freitas , et al., “GISAID's Role in Pandemic Response,” China CDC Weekly 3, no. 49 (2021): 1049–1051, 10.46234/ccdcw2021.255. 34934514 PMC8668406

[16] T. Burki , “First Shared SARS‐CoV‐2 Genome: GISAID vs virological.org,” Lancet Microbe 4, no. 6 (2023): e395, 10.1016/S2666-5247(23)00133-7. 37116518 PMC10129129

[17] K. Katoh and D. M. Standley , “MAFFT Multiple Sequence Alignment Software Version 7: Improvements in Performance and Usability,” Molecular Biology and Evolution 30, no. 4 (2013): 772–780, 10.1093/molbev/mst010. 23329690 PMC3603318

[18] H. Sobhy and P. Colson , “Gemi: PCR Primers Prediction From Multiple Alignments,” Comparative and Functional Genomics 2012 (2012): 783138, 10.1155/2012/783138. 23316117 PMC3535827

[19] J. Quick , N. D. Grubaugh , S. T. Pullan , et al., “Multiplex PCR Method for MinION and Illumina Sequencing of Zika and Other Virus Genomes Directly From Clinical Samples,” Nature Protocols 12, no. 6 (2017): 1261–1276, 10.1038/nprot.2017.066. 28538739 PMC5902022

[20] N. D. Grubaugh , K. Gangavarapu , J. Quick , et al., “An Amplicon‐Based Sequencing Framework for Accurately Measuring Intrahost Virus Diversity Using PrimalSeq and iVar,” Genome Biology 20, no. 1 (2019): 8, 10.1186/s13059-018-1618-7. 30621750 PMC6325816

[21] N. Papa Mze , I. Kacel , M. Beye , et al., “High Throughput SARS‐CoV‐2 Genome Sequencing From 384 Respiratory Samples Using the Illumina COVIDSeq Protocol,” Genes 14, no. 3 (2023): 681, 10.3390/genes14030681. 36980953 PMC10048438

[22] S. Kumar , K. Tamura , and M. Nei , “MEGA: Molecular Evolutionary Genetics Analysis Software for Microcomputers,” Bioinformatics 10, no. 2 (1994): 189–191, 10.1093/bioinformatics/10.2.189. 8019868

[23] I. Letunic and P. Bork , “Interactive Tree of Life (iTOL): An Online Tool for Phylogenetic Tree Display and Annotation,” Bioinformatics 23, no. 1 (2007): 127–128, 10.1093/bioinformatics/btl529. 17050570

[24] I. Aksamentov , C. Roemer , E. B. Hodcroft , and R. A. Neher . “Nextclade: Clade Assignment, Mutation Calling and Quality Control for Viral Genomes,” Journal of Open Source Software 6, no. 67 (2021): 3773, https://joss.theoj.org/papers/10.21105/joss.03773.

[25] P. Colson , H. Chaudet , J. Delerce , et al., “Role of SARS‐CoV‐2 Mutations in the Evolution of the COVID‐19 Pandemic,” Journal of Infection 88, no. 5 (2024): 106150, 10.1016/j.jinf.2024.106150. 38570164

[26] H. M. Berman , “The Protein Data Bank,” Nucleic Acids Research 28, no. 1 (2000): 235–242, 10.1093/nar/28.1.235. 10592235 PMC102472

[27] J. Fantini , N. Yahi , F. Azzaz , and H. Chahinian , “Structural Dynamics of SARS‐CoV‐2 Variants: A Health Monitoring Strategy for Anticipating Covid‐19 Outbreaks,” Journal of Infection 83, no. 2 (2021): 197–206, 10.1016/j.jinf.2021.06.001. 34089757 PMC8172274

[28] F. Azzaz , D. Hilaire , and J. Fantini , “Structural Basis of Botulinum Neurotoxin Serotype A1 Binding to Human SV2A or SV2C Receptors,” Chemico‐Biological Interactions 373 (2023): 110384, 10.1016/j.cbi.2023.110384. 36754227

[29] M. U. Johansson , V. Zoete , O. Michielin , and N. Guex , “Defining and Searching for Structural Motifs Using DeepView/Swiss‐PdbViewer,” BMC Bioinformatics 13, no. 1 (2012): 173, 10.1186/1471-2105-13-173. 22823337 PMC3436773

[30] P. Colson , P. E. Fournier , J. Delerce , et al., “Culture and Identification of a “Deltamicron” SARS‐CoV‐2 in a Three Cases Cluster in Southern France,” Journal of Medical Virology 94, no. 8 (2022): 3739–3749, 10.1002/jmv.27789. 35467028 PMC9088576

[31] P. Colson , B. La Scola , M. Beye , J. Delerce , D. Raoult , and J. Fantini , “Emergence of a Second SARS‐CoV‐2 Variant With a Tremendous Genetic Leap From Its Ancestors,” Journal of Medical Virology 95, no. 10 (2023): e29124, 10.1002/jmv.29124. 37811585

[32] J. Fantini , D. Carlus , and N. Yahi , “The Fusogenic Tilted Peptide (67–78) of α‐Synuclein Is a Cholesterol Binding Domain,” Biochimica et Biophysica Acta (BBA) ‐ Biomembranes 1808, no. 10 (2011): 2343–2351, 10.1016/j.bbamem.2011.06.017. 21756873

[33] P. V'kovski , A. Kratzel , S. Steiner , H. Stalder , and V. Thiel , “Coronavirus Biology and Replication: Implications for SARS‐CoV‐2,” Nature Reviews Microbiology 19, no. 3 (2021): 155–170, 10.1038/s41579-020-00468-6. 33116300 PMC7592455

[34] X. Ou , H. Guan , B. Qin , et al., “Crystal Structure of the Receptor Binding Domain of the Spike Glycoprotein of Human Betacoronavirus HKU1,” Nature Communications 8, no. 1 (2017): 15216, 10.1038/ncomms15216. PMC5529671 28534504

[35] N. Shao , C. Zhang , J. Dong , et al., “Molecular Evolution of Human Coronavirus‐NL63, ‐229E, ‐HKU1 and ‐OC43 in Hospitalized Children in China,” Frontiers in Microbiology 13 (2022): 1023847, 10.3389/fmicb.2022.1023847. 36406425 PMC9666422

[36] M. McCallum , Y. J. Park , C. Stewart , et al., “Human Coronavirus HKU1 Recognition of the TMPRSS2 Host Receptor,” Cell 187, no. 16 (2024): 4231–4245.e13, 10.1016/j.cell.2024.06.006. 38964328 PMC12854727

[37] N. Zhu , D. Zhang , W. Wang , et al., “A Novel Coronavirus From Patients With Pneumonia in China, 2019,” New England Journal of Medicine 382 (2020): 727–733, 10.1056/NEJMoa2001017. 31978945 PMC7092803

[38] Q. Li , T. Shah , B. Wang , et al., “Cross‐Species Transmission, Evolution and Zoonotic Potential of Coronaviruses,” Frontiers in Cellular and Infection Microbiology 12 (2023): 1081370, 10.3389/fcimb.2022.1081370. 36683695 PMC9853062

[39] R. Z. Ye , C. Gong , X. M. Cui , et al., “Continuous Evolution and Emerging Lineage of Seasonal Human Coronaviruses: A Multicenter Surveillance Study,” Journal of Medical Virology 95 (2023): e28861, 10.1002/jmv.28861. 37310144

[40] M. G. Mah , M. A. Zeller , R. Zhang , et al., “Discordant Phylodynamic and Spatiotemporal Transmission Patterns Driving the Long‐Term Persistence and Evolution of Human Coronaviruses,” NPJ Viruses 2 (2024): 49. 10.1038/s44298-024-00058-w PMC11721344 40295720

[41] A. Giraud‐Gatineau , L. Kaba , C. Boschi , et al., “Control of Common Viral Epidemics but Not of SARS‐CoV‐2 Through the Application of Hygiene and Distancing Measures,” Journal of Clinical Virology 150–151 (2022): 105163, 10.1016/j.jcv.2022.105163. PMC9013017 35472752

[42] T. Sloots , P. McErlean , D. Speicher , K. Arden , M. Nissen , and I. Mackay , “Evidence of Human Coronavirus HKU1 and Human Bocavirus in Australian Children,” Journal of Clinical Virology 35, no. 1 (2006): 99–102, 10.1016/j.jcv.2005.09.008. 16257260 PMC7108338

[43] X. Chen , Y. Zhu , Q. Li , et al., “Genetic Characteristics of Human Coronavirus HKU1 in Mainland China During 2018,” Archives of Virology 167, no. 11 (2022): 2173–2180, 10.1007/s00705-022-05541-4. 35840864 PMC9287133

[44] S. R. Dominguez , S. Shrivastava , A. Berglund , et al., “Isolation, Propagation, Genome Analysis and Epidemiology of HKU1 Betacoronaviruses,” Journal of General Virology 95, no. 4 (2014): 836–848, 10.1099/vir.0.059832-0. 24394697 PMC3973476

[45] J. Fantini , H. Chahinian , and N. Yahi , “Convergent Evolution Dynamics of SARS‐CoV‐2 and HIV Surface Envelope Glycoproteins Driven by Host Cell Surface Receptors and Lipid Rafts: Lessons for the Future,” International Journal of Molecular Sciences 24, no. 3 (2023): 1923, 10.3390/ijms24031923. 36768244 PMC9915253

[46] M. F. Pronker , R. Creutznacher , I. Drulyte , et al., “Sialoglycan Binding Triggers Spike Opening in a Human Coronavirus,” Nature 624, no. 7990 (2023): 201–206, 10.1038/s41586-023-06599-z. 37794193 PMC10700143

[47] D. Hammache , N. Yahi , M. Maresca , G. Piéroni , and J. Fantini , “Human Erythrocyte Glycosphingolipids as Alternative Cofactors for Human Immunodeficiency Virus Type 1 (HIV‐1) Entry: Evidence for CD4‐Induced Interactions Between HIV‐1 gp120 and Reconstituted Membrane Microdomains of Glycosphingolipids (Gb3 and GM3),” Journal of Virology 73, no. 6 (1999): 5244–5248, 10.1128/JVI.73.6.5244-5248.1999. 10233996 PMC112578

[48] M. Matveeva , M. Lefebvre , H. Chahinian , N. Yahi , and J. Fantini , “Host Membranes as Drivers of Virus Evolution,” Viruses 15, no. 9 (2023): 1854, 10.3390/v15091854. 37766261 PMC10535233

