# Supplementary material for: Five‐Year (2017–2022) Evolutionary Dynamics of Human Coronavirus HKU1 in Southern France With Emergence of Viruses Harboring Spike H512R Substitution
Source: J Med Virol. 2025 Feb 14;97(2):e70217. doi: 10.1002/jmv.70217 (PMC11826117; doi:10.1002/jmv.70217)
Supplement: Supplementary file 1 — Supporting information. [file JMV-97-e70217-s001.docx]

**Supplementary material**

**for**

Five-year (2017-2022) evolutionary dynamics of human coronavirus HKU1 in southern France with emergence of viruses harboring spike H512R substitution

**Author list: Houmadi Hikmat^1,2^, Lorlane Le Targa^1,2,3^, Céline Boschi^1,2,4^, Justine Py^1,2^, Aurélie MORAND^4,5,6^, Jean-Christophe LAGIER^1,2,4^, Sarah Aherfi^1,2,4^, Jacques FANTINI^7^, Bernard La Scola^1,2,4*^, Philippe Colson^1,2,4*^**

**Affiliations:** ^1^ Microbes Evolution Phylogeny and Infections (MEPHI), Aix-Marseille Univ. (AMU), Marseille, France; ^2^ IHU Méditerranée Infection, 19-21 boulevard Jean Moulin, 13005 Marseille, France; ^3^ Biosellal, 27 Chemin des Peupliers, 69570 Lyon, France; ^4^ Assistance Publique-Hôpitaux de Marseille (AP-HM), Marseille, France; ^5^ Service d'accueil des Urgences Pédiatriques, hôpital Nord, AP-HM, chemin des Bourrely, 13015 Marseille, France; ^6^ Service de Pédiatrie générale, hôpital Timone, AP-HM, 264 rue Saint-Pierre, 13005 Marseille, France; ^7^ INSERM UMR UA 16, Aix-Marseille Université, Marseille, France

**Supplementary methods**

We used the same methodology than in a previous study ^1^ to classify genes into “structural”, “informational”, “other non-structural” and “accessory” genes. In the structural gene category, we included the genes encoding the hemagglutinin esterase and the N2 part of the nucleocapsid, which are absent in SARS-CoV-2 but are present in HCoV-HKU1, and in the accessory gene category we only included the ORF4 gene. In order to analyze three particular mutations in the spike, we retrieved the full-length spike amino acid sequences from the NCBI GenBank protein sequence database using "HCoV-HKU1” and “Spike“ as keywords, and analyzed them concurrently with those obtained in the present study.

**Supplementary results**

**HCoV-HKU1 mutational patterns**

***Genotype A sequences***

Compared to reference genome LC315650.2 dating back to 2014, a total of 5,671 nucleotide substitutions, 417 deletions and 855 insertions were observed in the genomes obtained here. The gene that showed the greatest number of nucleotide substitutions relatively to its length was nsp9, which encodes a single-stranded DNA/RNA-binding protein ^2^, followed by nsp4, which was involved in deflecting host endomembranes into replication organelles ^3^, with 394 and 392 substitutions per 1,000 nucleotides, respectively; both these genes are part of the category of non-structural genes. In this category, the gene that showed the lowest number of nucleotide substitutions was nsp1, which encodes a host shutoff protein that controls anti-viral responses ^4^, with 112 substitutions per 1,000 nucleotides. Among informational genes, as relatively to the 2004 reference, nsp12 was also the gene that exhibited the greatest number of nucleotide substitutions (339 substitutions per 1,000 nucleotides), and nsp15 exhibited the lowest number of nucleotide substitutions in this category (48 per 1,000 nucleotides). The HE encoding gene and the spike gene were the structural genes with the greatest number of mutations with 302 and 182 substitutions per 1,000 nucleotides, respectively, while the envelope gene carried the lowest number of nucleotides substitutions (12 per 1,000 nucleotides) (Supplementary Table S3). The accessory genes carried the same prevalence of susbstitutions (57 per 1,000 nucleotides) as relatively to the reference genome dating back to 2004.

Regarding amino acid mutations relatively to the reference genome LC315650.2 dating back to 2014, 1,629 amino acid substitutions were found. The number of amino acid substitutions ranged from 14 to 319 with a mean value of 55.0±46.1; the number of deletions ranged from 0 to 38 with a mean value of 9.0±8.7; and the number of insertions ranged from 1 to 42 with a mean value of 4.7±6.0. A total of 1,629 amino acid substitutions were detected. Among informational proteins, nsp12 was the protein with the greatest diversity with 299 amino acid substitutions per 1,000 amino acids; nsp15 exhibited the lowest diversity with 42 substitutions per 1,000 amino acids. Among structural proteins, HE followed by spike showed the greatest diversity with 290 and 144 substitutions per 1,000 amino acids, respectively, and envelope showed the lowest diversity (Supplementary Table S3). Of these 1,629 amino acid substitutions, only 41 were present in at least 10 genomes (Supplementary Figure S1A). The spike was the protein harboring the greatest number of substitutions. The proteins with no mutation present in at least 10 genomes were nsp4, nsp5, nsp6, nsp10, nsp11, nsp13, M, N2, E and ORF4. Interestingly, two stop codons (N193* in nsp4, and C1322* in the spike gene) were present in 11 and 10 of the genomes recovered here, respectively.

***Genotype B sequences***

A total of 6,149 nucleotide substitutions, 428 deletions and 789 insertions were detected in the genotype B sequences obtained in this study relatively to the reference genome LC315651.2 dating back to 2016. We observed results similar to those comparing these genomes to the older (dating back to 2005) genotype B reference genome (Supplementary Table S5). In comparison with this reference, genomes obtained here encoded a total of 1,789 amino acid susbtitutions. The number of amino acid substitutions ranged from 18 to 243 with a mean value of 56.7±44.6. The number of deletions was between 1 and 47 with an average of 9.2±9.5 and the number of insertions was between 0 and 35 with a mean value of 2.7±5.1. Of them, 61 were only present in at least 10 genomes. The spike was the protein with the greatest number of mutations. The proteins that harbored no amino acid substitution present in at least 10 genomes were nsp1, nsp5, nsp6, nsp8, nsp10, nsp11, nsp13, nsp15, nsp16, and ORF4 (Supplementary Figure S1B). Finally, in these genotype B genomes, three stop codons were detected, including L6* in the HE, F171* in nsp15, and N193* in nsp4, in 19, 14 and 10 of these genomes, respectively.

**Supplementary figures**

**Supplementary Figure S1: Frequencies of mutations in genotype A (a) and genotype B (b) genomes compared with reference genomes LC315650.2 and LC315651.2, respectively**

**a.**

**b.**

**SupplemenTAry tables**

**Supplementary Table S1. Sequences of primers used for PCR amplifcation prior to the next-generation sequencing of the HCoV-HKU1 genomes**

| **Primer name** | **Sequence (5'-3' orientation)** | **Pool** | **Concentration of use (pmol/µL)** |
| --- | --- | --- | --- |
| HKU1_F1 | ACGATCTCTTGTCAGATC**Y**C | 1 | 15.0 |
| HKU1_R1 | CAATCTACATAACCATACTGCA |  | 15.0 |
| HKU1_F2 | GTGGTTTTGTTATGCAGTATG | 2 | 15.0 |
| HKU1_R2 | GGTTGACAATACTCATAAGATG |  | 15.0 |
| HKU1_F3 | TTTATTTTTTTGAAGGCGTTGCA | 1 | 15.0 |
| HKU1_R3 | GTCACCAGTAACAACATCTTC |  | 15.0 |
| HKU1_F4_ALT | ACTGGTGACAATGACGATGA | 2 | 12.6 |
| HKU1_R4 | CATACA**R**TTGAG**S**TAACTCAAA |  | 12.6 |
| HKU1_F5 | CTGTGGATGTAAACGTTTGTC | 1 | 10.0 |
| HKU1_R5 | GC**R**AGCAATTGTTGCTGATC |  | 10.0 |
| HKU1_F6 | GGTAATGTTTTCTGTGATGGC | 2 | 10.0 |
| HKU1_R6 | CAACCCAAACAACATAATCACA |  | 10.0 |
| HKU1_F7 | GTATCTGAGGAGTCACAAGG | 1 | 10.0 |
| HKU1_R7 | AGGTCGTTTAAGCTCTTTAGA |  | 10.0 |
| HKU1_F8 | TCTAAAGAGCTTAAACGACCT | 2 | 10.0 |
| HKU1_R8 | GTGGAAC**Y**AAAGATGTATACAA |  | 10.0 |
| HKU1_F9 | AAGAGGTGATGGTACACCAC | 1 | 10.0 |
| HKU1_R9 | CATAACCAACAGATCCACATG |  | 10.0 |
| HKU1_F10 | GCATTTCATGTTACTATGCGTA | 2 | 15.0 |
| HKU1_R10 | CTACAATACTGCCACAAC**Y**TA |  | 15.0 |
| HKU1_F11_ALT | GAATAGGTGGCGTGCCAGT | 1 | 12.6 |
| HKU1_R11_ALT | ACAGACTTGACAAACATCATGT |  | 12.6 |
| HKU1_F12 | CGTGTAGAGCAT**Y**CAGATGT | 2 | 10.0 |
| HKU1_R12 | AAATCAACTGTACTACCCTCTT |  | 10.0 |
| HKU1_F13 | GTAAAGGCTTGTTTAAAGAGG | 1 | 12.6 |
| HKU1_R13 | ACTATCTAAGATCTGA**K**TACCA |  | 12.6 |
| HKU1_F14 | GAGCGCTTTGTAAGTCTAGC | 2 | 10.0 |
| HKU1_R14_ALT | CCCAAAAA**R**ATATCAGGACCT |  | 10.0 |
| HKU1_F15 | GTGCT**R**TTGAG**Y**AAGGGTTC | 1 | 12.6 |
| HKU1_R15 | GCAACATGTGCACCTTTATGT |  | 12.6 |
| HKU1_F16 | TTGTAGATATACAACAGTGGG | 2 | 10.0 |
| HKU1_R16 | CATCGTCACCATCTTTTCTCA |  | 10.0 |
| HKU1_F17 | GTCC**R**CAACGAGCTGATTTA | 1 | 10.0 |
| HKU1_R17 | ACC**R**CCATTCCATGTTGTAC |  | 12.6 |
| HKU1_F18 | AAATGCTTCTTCTAGTGAAGG | 2 | 15.0 |
| HKU1_R18 | AATCAACAACCTG**Y**AC**R**GGTA |  | 15.0 |
| HKU1_F19 | TACACTGGCGA**R**GGTGATC | 1 | 10.0 |
| HKU1_R19 | CACCAG**M**TTTAGG**R**AAATAAC |  | 10.0 |
| HKU1_F20 | GCCTACAACA**Y**TAGCTGTTAT | 2 | 15.0 |
| HKU1_R20 | CAATTAAGAGGTGA**R**GGTACA |  | 15.0 |
| HKU1_F21 | CTGT**W**AAGCCTGTTGCAACT | 1 | 15.0 |
| HKU1_R21 | ATT**R**GAACAAGTGGT**R**CCAC |  | 15.0 |
| HKU1_F22 | AGTGG**Y**ACCACTTGTTC**Y**AAT | 2 | 15.0 |
| HKU1_R22 | CACTTTG**W**ATTTTAGCAAG**W**GC |  | 15.0 |
| HKU1_F23 | AATGGTTTGGGTGTTACTATG | 1 | 15.0 |
| HKU1_R23 | GACATTTACTAAAACATGC**W**GA |  | 15.0 |
| HKU1_F24 | TGGTTG**Y**GGTTC**W**GCATGTT | 2 | 15.0 |
| HKU1_R24 | GGCCTAACA**W**ACATCTT**R**CC |  | 15.0 |
| HKU1_F25 | GAACTGG**M**AGTTGGTGGAG | 1 | 10.0 |
| HKU1_R25 | TCA**R**ATTGGTCA**R**CCCAAGAA |  | 10.0 |
| HKU1_F26 | GTCAT**Y**ATGCTGGAAGTAGAA | 2 | 10.0 |
| HKU1_R26 | TGAGAACGTGAACCTGG**Y**C |  | 10.0 |
| HKU1_F27 | TACGATT**Y**TGCCTCAAGGCT | 1 | 10.0 |
| HKU1_R27 | CTTTCATAATTGTCTCAAA**R**CC |  | 10.0 |
| HKU1_F28 | TACTTTACC**W**GG**Y**TTTGAGAC | 2 | 15.0 |
| HKU1_R28 | CAGCTAAACCTTATAACTACTC |  | 15.0 |

Degenerated nucleotide positions are indicated by a bold font.

**Supplementary Table S2. Frequency of nucleotide and amino acid substitutions per 1,000 nucleotides compared with the genotype A reference genome (NC_006577.2)**

**Supplementary Table S3. Frequency of nucleotide and amino acid substitutions per 1,000 nucleotides compared with the genotype A reference genome LC315650.2**

**Supplementary Table S4. Frequency of nucleotide and amino acid substitutions per 1,000 nucleotides compared with the genotype B reference genome DQ415911.1**

**Supplementary Table S5. Frequency of nucleotide and amino acid substitutions per 1000 nucleotides compared with the genotype B reference genome (LC315651.2)**

**REFERENCES**

1. Colson P, Chaudet H, Delerce J, Pontarotti P, Levasseur A, Fantini J, et al. Role of SARS-CoV-2 mutations in the evolution of the COVID-19 pandemic. *J Infect* 2024;88(5):106150. Doi: 10.1016/j.jinf.2024.106150.

2. V’kovski P, Kratzel A, Steiner S, Stalder H, Thiel V. Coronavirus biology and replication: implications for SARS-CoV-2. *Nat Rev Microbiol* 2021;19(3):155–70. Doi: 10.1038/s41579-020-00468-6.

3. Liu Ding X., Liang Jia Q., Fung To S. Human Coronavirus-229E, -OC43, -NL63, and -HKU1 (Coronaviridae). In: Bamford Dennis H., Zuckerman Mark, editors. *Encycl. Virol. Fourth Ed.* Oxford: Academic Press; 2021. pp. 428–40.

4. Bermudez Y, Miles J, Muller M. Nonstructural protein 1 widespread RNA decay phenotype varies among coronaviruses. *iScience* 2023;26(1). Doi: 10.1016/j.isci.2022.105887.
